# Early Detection of Freezing of Gait during Walking Using Inertial Measurement Unit and Plantar Pressure Distribution Data

**DOI:** 10.3390/s21062246

**Published:** 2021-03-23

**Authors:** Scott Pardoel, Gaurav Shalin, Julie Nantel, Edward D. Lemaire, Jonathan Kofman

**Affiliations:** 1 Department of Systems Design Engineering, University of Waterloo, Waterloo, ON N2L 3G1, Canada; spardoel@uwaterloo.ca (S.P.); gshalin@uwaterloo.ca (G.S.); 2 School of Human Kinetics, Faculty of Health Sciences, University of Ottawa, Ottawa, ON K1N 6N5, Canada; jnantel@uottawa.ca; 3 Faculty of Medicine, University of Ottawa, Ottawa Hospital Research Institute, Ottawa, ON K1H 8M2, Canada; elemaire@ohri.ca

**Keywords:** Parkinson’s disease, freezing of gait, wearable sensors, detection, prediction, machine learning

## Abstract

Freezing of gait (FOG) is a sudden and highly disruptive gait dysfunction that appears in mid to late-stage Parkinson’s disease (PD) and can lead to falling and injury. A system that predicts freezing before it occurs or detects freezing immediately after onset would generate an opportunity for FOG prevention or mitigation and thus enhance safe mobility and quality of life. This research used accelerometer, gyroscope, and plantar pressure sensors to extract 861 features from walking data collected from 11 people with FOG. Minimum-redundancy maximum-relevance and Relief-F feature selection were performed prior to training boosted ensembles of decision trees. The binary classification models identified Total-FOG or No FOG states, wherein the Total-FOG class included data windows from 2 s before the FOG onset until the end of the FOG episode. Three feature sets were compared: plantar pressure, inertial measurement unit (IMU), and both plantar pressure and IMU features. The plantar-pressure-only model had the greatest sensitivity and the IMU-only model had the greatest specificity. The best overall model used the combination of plantar pressure and IMU features, achieving 76.4% sensitivity and 86.2% specificity. Next, the Total-FOG class components were evaluated individually (i.e., Pre-FOG windows, Freeze windows, transition windows between Pre-FOG and Freeze). The best model detected windows that contained both Pre-FOG and FOG data with 85.2% sensitivity, which is equivalent to detecting FOG less than 1 s after the freeze began. Windows of FOG data were detected with 93.4% sensitivity. The IMU and plantar pressure feature-based model slightly outperformed models that used data from a single sensor type. The model achieved early detection by identifying the transition from Pre-FOG to FOG while maintaining excellent FOG detection performance (93.4% sensitivity). Therefore, if used as part of an intelligent, real-time FOG identification and cueing system, even if the Pre-FOG state were missed, the model would perform well as a freeze detection and cueing system that could improve the mobility and independence of people with PD during their daily activities.

## 1. Introduction

Freezing of gait (FOG) is an intermittent walking disturbance common in the more advanced stages of Parkinson’s disease (PD) and is characterized by an inability to move the feet, often with the sensation of having one’s feet glued to the ground [1]. Sudden and often unexpected FOG episodes can lead to falling and fall-related injuries that can have severe health repercussions [2,3].

Auditory, visual, and tactile cues can help a person overcome freezing episodes and resume walking [4,5]. Freeze-detection systems to automatically identify a freeze episode and activate an assistive cue only when needed are increasingly being studied [4]. However, while cueing systems based on FOG detection can reduce freezing episode duration, the risk of falling due to freezing is still present because the cue is only administered after freeze onset. A practical freeze identification and cueing system should detect freezing as early as possible. Ideally, oncoming freeze episodes would be predicted, and a pre-emptive cue would be used to prevent the episode. If prediction is not possible, early detection of FOG such that a cue can be administered immediately after freeze onset would also be beneficial. 

Various machine learning models using wearable-sensor data were developed to predict FOG by assuming the presence of distinct gait characteristics prior to the onset of a freezing episode and training a classifier to identify this pre-freeze state [6,7,8,9,10,11,12]. Recently, a long short-term memory (LSTM) neural network was trained with data from all but one participant. A final layer was trained only on the target participant’s data (only weights of the final layer were updated). This transfer learning model achieved over 90% accuracy [11]; however, accuracy may overestimate model performance since all correct classifications are used, regardless of class. Thus, a model that missed most FOG episodes but correctly classified all non-FOG data may still have high accuracy, especially if the non-FOG class greatly outnumbers FOG data, which is typically the case. FOG prediction has also been approached as a time series prediction problem, where autoregressive predictive models projected the feature time series [13], which was then classified as FOG or Non-FOG using support vector machines and probabilistic neural networks. This method achieved FOG prediction sensitivity and specificity above 90% [13]. However, FOG prediction in [11,13,14] used the Daphnet dataset [15] (237 FOG episodes), where the majority of participants were in the OFF antiparkinsonian medication state. PD-related movement symptoms can dramatically improve with medication and worsen as the medication wears off [16,17]. People with PD who would benefit most from FOG identification and cueing devices walk and perform activities of daily living independently and are likely on medication to enable this quality of life. Therefore, data collected while the participants are on their medication should be used to develop FOG identification systems. For the ON medication state, gait parameters are less abnormal compared to OFF medication state [18]. Thus, gait characteristics associated with FOG (or imminent FOG) may be less pronounced for ON medication state. Investigation is still needed to determine the best combination of algorithm and sensor type for FOG detection and prediction, especially for the ON medication state.

Inertial measurement units (IMUs) on the lower limbs have been used for FOG classification [6,12,14,19,20,21]. In [14], the shank was the preferred sensor location for FOG episode prediction, and in [19], a model with ankle accelerometer data predicted 66.7% of the FOG episodes within 2 s prior to onset. To improve FOG identification performance, additional sensor types could be used. Since a complex interaction exists between postural stability and freezing [16], plantar pressure sensors may detect subtle parameters linked to FOG (e.g., weight transfer changes between feet or foot centre of pressure movement [22]) that would be difficult to detect using IMUs. Plantar pressure distribution and ground reaction forces have been used for FOG detection [23,24] and for a variety of gait and balance studies in PD populations [25,26,27]. Plantar pressure analysis has also been used in post-traumatic rehabilitation [28], stroke rehabilitation [29], fall-risk prediction [30], faller classification [31], and classifying individuals as PD or a healthy control from walking data [32]. Furthermore, the pressure distribution may vary distinctly between phases of normal walking, during transition from normal walking into freeze, and during a freeze. Preliminary research using plantar pressure data for FOG detection and prediction has shown promise [33,34]; thus, plantar pressure analysis may open new avenues in predicting FOG events. 

This research determined the effectiveness of FOG detection and prediction models based on plantar pressure and IMU data, used separately and together. Since the ultimate goal was to develop a real-time FOG prediction system that could enable preventative cueing, a computationally-light decision-tree-based classification model was developed and evaluated. Notably, the model was trained with grouped Pre-FOG, Pre-FOG transition (windows containing both Pre-FOG and FOG data), and FOG data and then evaluated using these data labels grouped and separately. Participants were on their normal antiparkinsonian medication schedule and dosage, to permit a somewhat realistic medication condition. The developed models would ultimately be used to detect FOG as early as possible and thereby become part of a novel wearable intelligent-cueing system. If FOG classification models using plantar pressure sensors are found to be viable for FOG classification, this could lead to a self-contained in-shoe device that could have high user compliance and provide FOG mitigation with a user-friendly wearable system.

## 2. Materials and Methods

### 2.1. Data Collection

**Figure 1:**
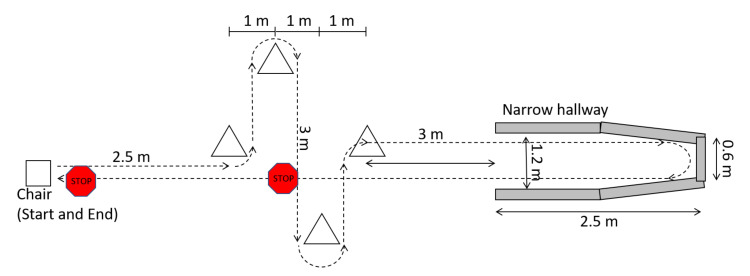
Experiment walking path.

Eleven male participants with PD were recruited from the community. Participants were required to experience freezing at least once a week, be able to walk unassisted, not have undergone deep brain stimulation therapy, and not have balance or mobility conditions (other than PD) that affect walking. Participants visited the lab for a single data collection session while on their normal antiparkinsonian medication dosage and schedule. Data collection was typically scheduled in the hours prior to the participant’s next dose so that the medication would be wearing off during testing and FOG would be more likely to occur. Ethics approval was obtained from the University of Ottawa (H-05-19-3547) and University of Waterloo (40954), and all participants provided informed written consent. Participants were asked to walk a complex freeze-provoking path up to 30 times. The path started and ended in a seated position and included 90° and 180° turns, stops, starts, and a narrow passageway leading to a dead end (Figure 1).

While walking the path, participants were asked to perform additional tasks simultaneously to increase the likelihood of freezing. These tasks were both physical (holding a plastic tray with objects on top) and verbal (naming as many words as possible starting with a specific letter). Motor-task difficulty was increased if the participant did not find the task challenging. For example, the motor task started with three small wooden blocks on the tray, but additional blocks were added as needed to increase difficulty. Alternatively, the blocks were replaced with an empty paper coffee cup or a sealed water bottle, or the participant was asked to carry the tray with only one hand. In total, 241 min of walking data were collected, during which seven participants froze. Similar to [15] and [35], the beginning of a freeze was defined as “the instant the stepping foot fails to leave the ground despite the clear intention to step” and the end of the freeze was defined as “the instant the stepping foot begins or resumes an effective step”.

**Figure 2:**
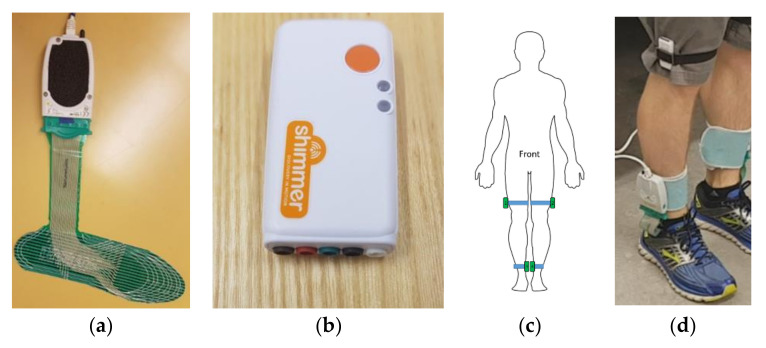
Sensor systems used in data collection: (**a**) FScan pressure-sensing insole, (**b**) Shimmer3 inertial measurement unit (IMU) sensor, (**c**) diagram of IMU placement, and (**d**) photograph of insole and IMU systems worn on body.

During the walking trials, plantar pressure data were collected using FScan pressure-sensing insoles (Tekscan, Boston, MA). The flexible insoles are less than 1 mm thick with 3.9 pressure-sensing cells per cm^2^ (Figure 2a). A new pair of insoles was used for each participant and trimmed to fit inside their regular shoes. The insoles were equilibrated prior to the participant data-collection session. At the beginning of data collection, the sensors were calibrated by asking the participant to stand with all their weight on a single foot and then shift to stand on the other foot. This was done for both feet.

In addition to the plantar pressure sensors, the Shimmer3 IMU system (Shimmer, Dublin, Ireland) was used to record lower limb acceleration and angular rotation (Figure 2b). A sensor was placed on the medial side of each shank, just above the malleolus, and lateral side of each thigh, just above the knee (Figure 2c,d). IMU data were collected at 512 Hz and downsampled in post-processing to match the plantar pressure sampling rate of 100 Hz. Walking trials were video-recorded using a smartphone camera for post-collection FOG identification. IMU, plantar pressure insole, and video signals were synchronized using a single foot stomp performed at the beginning of each walking trial. 

### 2.2. Labelling and Windowing

FOG instances were identified visually from the video using a custom labelling program written using MATLAB R2019b App Designer (MathWorks, Natick, MA, USA). During data collection, authors SP and JN identified FOG occurrences. In post-processing, SP identified the onset and termination of FOG episodes using video data with a 30 Hz frame rate. In case of uncertainty, the second rater was consulted. During labelling, synchronization of IMU, plantar pressure insole, and video signals was confirmed using multiple heel-strike events. 

**Figure 3:**
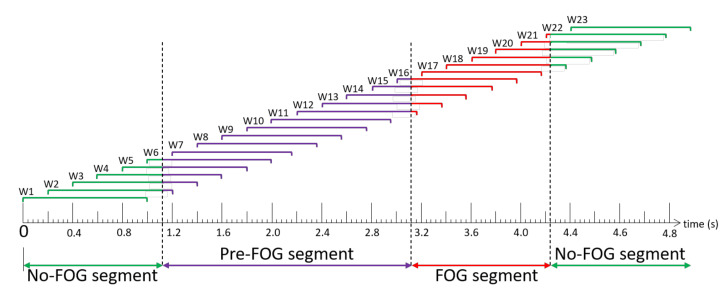
Freezing of gait (FOG) episode windowing scheme example. Windows (W) 1–6 are “No-FOG”, Windows 7–11 are “Pre-FOG”, Windows 12–16 overlap the Pre-FOG and FOG segments and are thus “Pre-FOG-Transition”, Window 17 is entirely in the FOG segment and is “FOG”, and Windows 18–23 extend or entirely occur beyond the end of the FOG episode and are “No-FOG”.

Data were windowed using a 1 s sliding window with a 0.2 s shift between windows (i.e., 0.8 s overlap between consecutive windows) (Figure 3). The Pre-FOG segment was defined as the 2 s period immediately before FOG episode onset. With reference to Figure 3, each window (W) was labelled as Pre-FOG (entire window within 2 s before a freeze (W7–W11)), FOG (entire window during the freeze (W17)), Pre-FOG-Transition (window containing both Pre-FOG and FOG instances (W12–W16)), or No-FOG (window without any freeze (W1, W23)), or window that includes No-FOG instances and overlaps with the beginning of Pre-FOG gait (W2–W6) or the end of FOG (W18–W22). Another combined label was generated as Total-FOG, which contained all Pre-FOG and FOG instances (Pre-FOG, Pre-FOG-Transition, FOG (W7–W17)).

### 2.3. Feature Extraction

The features used in this research were based on [34] (Table 1). In total, 861 individual features were extracted from the 71,067 data windows. Features were grouped by time domain (n = 13), fast Fourier transform (n = 8), and discrete wavelet transform (Haar mother wavelet) (n = 14). All features were calculated separately for the left and right sides, with the exception of “number of weight shifts” that required data from both feet. For the FFT and WT categories, 38 signal inputs were used: total ground reaction force (GRF); position, velocity, and acceleration of foot centre of pressure (COP) in Y (anterior/posterior (AP)) and X (medial/lateral (ML)) directions; ankle and thigh acceleration in anterior/posterior (X), vertical (Y), and medial/lateral (Z) directions; and ankle and thigh angular rotation in anterior/posterior (X), vertical (Y), and medial/lateral (Z) directions. COP velocity and acceleration were calculated as the first and second derivatives of COP position, respectively. A total of 528 features were calculated from accelerometer and gyroscope data, and 333 features were calculated from plantar pressure data (GRF; COP position, velocity, acceleration). Before calculating COP, GRF values less than 5% of the two-foot total were set to 0, since the limb was in swing and the small pressures were not relevant to FOG. 

### 2.4. Feature Selection

Feature selection was performed to reduce the number of features and to determine which sensors contributed the most useful features. Feature selection and subsequent model development were performed three times: first with only features extracted from plantar pressure data, second with only IMU sensor features, and finally with all features. 

For feature selection, both minimum-redundancy maximum-relevance (mRMR) and Relief‑F feature selection algorithms were used. mRMR is a multivariate approach that selects features such that mutual information between a feature and class is maximized, while pairwise information between features is minimized [41]. mRMR has been used for FOG detection [34,35]. Relief-F incorporates interactions between features [42] and has been used in activity monitoring situations with plantar pressure data collected during walking [36]. Both mRMR and Relief-F performed feature selection by ranking features. Relief-F was performed with *k* = 200 nearest neighbours and 2000 updates.

For feature selection, the target class was composed of all windows with the Total-FOG label (including all Pre-FOG, Pre-FOG-transition, and FOG windows), and the nontarget class contained the No-FOG windows. 

### 2.5. Ensemble Model Development

A decision-tree ensemble was used for window classification. The base decision trees were tested with maximum depths of 5 or 10 decision splits and with the top 5, 10, 15, 20, 25, 50, 75, and 100 features according to both the Relief-F and mRMR feature selection methods. The ensemble of trees used random undersampling (RUS)-boosting and a maximum of 100 learning cycles. In pilot testing, RUS-boosting performed better than bagging and AdaBoosting approaches. This is likely due to the dataset being highly imbalanced, which can negatively affect classifier performance. RUS-boosting randomly undersamples the majority class (nontarget class in this study) so that the number of samples matches the minority class. Note that undersampling is only done during model training and not during testing; therefore, class imbalance in the testing data is unaffected. 

Leave-one-freezer-out (LOFO) cross-validation was used to evaluate the models. The typical leave-one-out cross-validation trains a model using the data from all but one person and then tests the model using the held-out person’s data. In FOG classification studies, it is common for some individuals to experience FOG in normal living but not during the in-laboratory data collection. Thus, if a person who did not freeze during testing was held out as the test subject, the corresponding test data would be entirely from the No-FOG class. This is problematic since a model cannot truly be evaluated using data from only the negative class. In some studies, the model is assumed to have 100% sensitivity for these individuals [35,43]; however, this assumption can skew overall model performance results. The LOFO method avoids this issue since only participants who froze during data collection are involved with model testing, while participants who did not experience FOG are always included in the training set. 

Five test cases were used during LOFO analysis (Table 2). The target and nontarget classes for the five test cases were defined as different groupings of the labelled windows. For each cross-validation fold, the model was trained only once using Case 1 (target class: Total-FOG, nontarget class: No-FOG) and then evaluated on each of the five test cases. Case 1, where the target class was Total-FOG windows and included Pre-FOG, Pre-FOG-transition, and FOG windows, was based on the goal of a clinically relevant cueing system, where real-time cueing would be activated before or during a freeze. For Cases 2, 4, and 5, the target class contained a single label. This was done to evaluate the model’s ability to recognize each of the labels individually. For Case 3, the Pre-FOG and Pre-FOG-Transition windows were grouped to form the target class, to examine the feasibility of using these two labels in future model development. This target class (Case 3) contained windows from the beginning of Pre-FOG data until, at most, 1 s into the FOG event; therefore, detection of windows in this target class would be either prediction of a freeze or detection of freeze episode initiation. Episode initiation detection would be useful in an intelligent cueing system.

## 3. Results

Table 3 presents participant information and the total number of windows of each label from each participant. Table 4 presents the LOFO cross-validation results for the three groups of features: plantar pressure features, IMU features, and both plantar pressure and IMU (PP-IMU).

Performance was very similar for the plantar pressure features model (sensitivity 78.0%, specificity 83.2%) and the PP-IMU features model (sensitivity 76.4%, specificity 86.2%) (Table 4). The IMU features model had the lowest sensitivity (61.9%) but the highest specificity (91.6%).

The ideal number of features and best feature ranking method differed for each group of features (Table 4). The best plantar pressure features model used the top 5 Relief-F features. The best IMU features model used the top 25 mRMR features. The best PP-IMU features model used the top 10 features according to Relief-F rankings. For all models, decision trees with 5 splits outperformed decision trees with 10 splits. The best model from Table 4 used combined features (i.e., PP-IMU features model), with 76.4% sensitivity and 86.2% specificity for the Total-FOG target class (Pre-FOG, Pre-FOG-Transition, FOG). For completeness, the nonfreezers were held out as test participants and the specificity was calculated (Table 4). The features used in the PP-IMU model are presented in Table 5.

The results for Cases 2–5 are presented in Table 6, Table 7 and Table 8. The specificity results for Cases 2–5 are constant across cases, since specificity is based on the nontarget class (true negatives and false positives), which is unchanged across cases.

## 4. Discussion

The research outcomes indicate that a decision-tree ensemble classifier using features from IMU and plantar pressure data together can appropriately identify Total-FOG (Pre-FOG, FOG Transition, FOG). This could lead to a wearable system where appropriate cues are provided to either avoid a freeze or help exit the freeze episode. The use of a decision-tree model will also facilitate integration with a real-time cueing system due to the low computational cost for this machine learning model.

Participants in this research were on their normal antiparkinsonian medication dosage and schedule. This is important since, in practice, FOG detection and cueing systems are for persons with PD who are taking medication to manage their motor-related symptoms to live independently.

Comparing the different models in Table 4 and the same test cases across Table 7 and Table 8, the plantar pressure features model reached higher sensitivity than the IMU features model. However, the IMU features model achieved higher specificity for all cases. This indicates that plantar pressure may identify FOG-related patterns that the IMU sensors cannot; however, plantar pressure sensors may produce more false positives. Thus, including features from both sensor systems is recommended.

The PP-IMU features model was selected as the best overall model. Further analysis from the additional four test cases (Table 6) showed that just over half the Pre-FOG windows were correctly identified. If this model were used to trigger an assistive cue, identifying 55.2% of the Pre-FOG windows before the FOG occurs would be helpful but may result in many missed opportunities to avoid a freeze (i.e., assuming that an appropriate cue can mitigate or avoid an upcoming freeze episode). For Pre-FOG-Transition, sensitivity was 85.2% using plantar pressure and IMU data, indicating that most transition windows between Pre-FOG and the freeze would be identified; therefore, a cue could be administered within the first second of the FOG episode. When Pre-FOG and Pre-FOG-Transition windows were combined, model sensitivity decreased to 70.2%. Hence, including Pre-FOG adversely affected freeze-event recognition. FOG window classification using plantar pressure and IMU data was highly effective (93.4% sensitivity), indicating that few FOG windows were missed. In practice, the freeze identification model would perform very well as a FOG detection system, with a cue administered during the freeze if the Pre-FOG or transition states were missed. A similar analysis in [19] predicted 66.7% of the freeze episodes within 2 s of onset and detected 97.4% of the episodes between 2 s before and 4 s after FOG onset. These results were based on the number of FOG episodes, which may account for the higher performance compared to results presented in this paper, where results were based on decisions for each window. 

PP-IMU features model sensitivity was 76.4%, indicating that approximately 24% of the target-class windows were missed by the model. Other FOG prediction research [13] reported higher sensitivity (93%), although as in [11,19], the performance metrics were calculated based on FOG episodes. Thus, the sensitivity results are not directly comparable to our window-based analysis. Furthermore, the method presented here is a participant-independent model. Typically, models that are adapted to a single individual perform better than those that are not user-specific. For instance, [19] tested both participant-independent and participant-dependent models and found better FOG prediction results with the person-specific models. For everyday wearable cueing devices, personalized freeze prediction systems are ideal. However, when validating new models, methods, or features, personalized models may not generalize well. Using personalized models and averaging across many participants could provide a better representation of model performance. Unfortunately, the datasets used in FOG prediction and detection studies are generally small. Moreover, individualized models usually require a large amount of data for each participant and are difficult to obtain.

PP-IMU features model specificity was 86.2%, indicating that approximately 14% of the nontarget classifications were false positives. In an intelligent cueing device, this could result in excessive false cues during walking, which may lead to reduced compliance, depending on the type of cue. To ensure that the cueing system is effective and is used as intended, the number of false cues could be minimized in future research on the cueing approach. For example, a decision threshold could be implemented such that consecutive classifications are required to trigger a cue. In addition, minimalistic or variable cues could be used such that false positives are better tolerated by the user. For instance, cue intensity or magnitude could begin at an almost imperceptible level and increase with successive positive FOG predictions. While 90% or greater specificity would be ideal, specificity below this threshold is common in the FOG prediction literature. Specificities of 67.0% [21], 80.25% [44], and recently 86% [45] have been reported.

The research outcomes could be applicable to a wearable freeze-detection system that is localized to the shoe. PP-IMU features model performance was only slightly better than the plantar pressure features model. While improvements could be made to plantar pressure features model sensitivity, the plantar pressure model performed very well as a detection system, detecting 98.5% of the FOG windows. The inclusion of IMU features in the PP-IMU features model was primarily to improve specificity. If plantar pressure features model specificity could be improved by other means, such as model personalization, then the IMU sensors could be excluded. A plantar-pressure-only system would have less complex hardware and software, be easier to don and doff, and could be more practical for long-term movement monitoring. A self-contained in-shoe system could have better user compliance since the instrumented shoes can be worn in daily activities.

While this research provided promising results, some limitations should be recognized. Seven people froze during testing and were included in the analysis. More participants will help with model generalization and model personalization. For instance, a larger participant pool would allow a more complete understanding of FOG manifestations and analysis of different FOG subtypes, leading to FOG-subtype-specific models. To further improve model performance, model personalization such as using individual-specific Pre-FOG and window durations could also be implemented. 

## 5. Conclusions

Accelerometer, gyroscope, and plantar pressure sensors were viable wearable devices for a FOG identification system. The combination of accelerometer, gyroscope, and plantar pressure data gave the best results. The best decision-tree ensemble model was built using 10 features and achieved 76.4% sensitivity and 86.2% specificity when classifying 1 s windows of Total-FOG data (data from 2 s before FOG onset until the end of the FOG episode). This model detected the transition between Pre-FOG gait and FOG with 85.2% sensitivity, which is equivalent to detecting FOG less than 1 s after the freeze began. Furthermore, the FOG windows were detected with 93.4% sensitivity, indicating that few FOG windows were missed. 

If the best model was applied in a wearable cueing device that can help avoid or break out of a freeze, this system would have a 70.2% chance of identifying FOG before or within 1 s of FOG onset. If this transition phase was missed, the cue would be applied during the freeze in 93.4% of occurrences. While the model using both plantar pressure and IMU features to detect Total-FOG had 86% specificity (i.e., 14% false-positive rate, which is common in FOG prediction studies), higher specificity is preferred in practice. To address this, a cueing threshold could be implemented such that a cue is only triggered if multiple consecutive positive classifications are obtained. Future work could also include additional participants, model personalization to improve performance, and window length or Pre-FOG duration optimization. 

## Figures and Tables

**Table 1:** Features extracted from windowed data.

Feature	Feature Description	Source	Number of Input Parameters	Total Features
Time domain features (n = 13)				
Number, duration, length of COP reversals	Number, length, duration of centre of pressure (COP) path direction reversals per window (n = 3)	[36]	2	6
Number, duration, length of COP deviations	Number, length, duration of mediolateral COP deviations per window. Deviation is the first derivative of COP ML exceeding a threshold of ±0.5 mm/window (n = 3)	[36]	2	6
CV of COP position, velocity, acceleration	Anterior/posterior (AP) and medial/lateral (ML) coefficients of variation (CV) of COP position, velocity, and acceleration (n = 6)	[36]	2	12
Number of weight shifts	Number of times the majority of total GRF (>50%) changed foot (n = 1)	-	1	1
			**Category total**	**25**
**Fast Fourier transform (FFT) features (n = 8)**				
Total power in FFT signal	Power in FFT signal per window as sum of squared amplitude (n = 1)	[37]	38	38
Dominant frequency	Frequency bin with highest amplitude per window (n = 1)	[38]	38	38
Max, min, mean	Maximum, minimum, and mean amplitude of FFT signal (n = 3)	[38]	38	114
Power in locomotion, freeze bands	Power under FFT curve in locomotion band (0.5–3 Hz) and freeze band (3–8 Hz) (n = 2)	[39]	38	76
Freeze index	Ratio of power in freeze band (3–8 Hz) and locomotion band (0.5–3 Hz) (n = 1)	[39]	38	38
			**Category total**	**304**
**Discrete wavelet transform features (n = 14), Haar mother wavelet**				
Variance of coefficients	Variance of the detail and approximation coefficient vectors (n = 2)	[40]	38	76
Max, min, mean	Maximum, minimum, mean of detail and approximation coefficient vectors (n = 6)	[40]	38	228
Max, min, mean energy	Maximum, minimum, mean energy of detail and approximation coefficient vectors (n = 6)	[40]	38	228
			**Category total**	**532**

**Table 2:** Target and nontarget class composition for each test case.

	Target Class	Nontarget Class
**Case 1**	Total-FOG: Pre-FOG, Pre-FOG-Transition, FOG	No-FOG
**Case 2**	Pre-FOG	No-FOG
**Case 3**	Pre-FOG, Pre-FOG-Transition	No-FOG
**Case 4**	Pre-FOG-Transition	No-FOG
**Case 5**	FOG	No-FOG

**Table 3:** Clinical details of the participants: number of years since PD diagnosis, New Freezing of Gait questionnaire (NFOG-Q), Unified Parkinson’s Disease Rating Scale Section III (UPDRS III); and number of data windows of each label extracted from each participant.

Participant	Years Since Diagnosis	NFOG-Q	UPDRS III	Window Labels			
				Pre-FOG	Pre-FOG-Transition	FOG	No-FOG
P01	16	14	10	217	166	7	3721
P02	11	21	20	178	171	294	5188
P03	11	17	13	66	62	17	6884
P04	10	4	18	0	0	0	2635
P05	14	20	13	0	0	0	5331
P06	19	22	29	52	49	162	9368
P07	5	15	16	725	1303	766	6572
P08	12	17	20	75	126	84	4848
P09	10	18	18	44	30	5	6848
P10	2	4	15	0	0	0	6034
P11	5	19	20	0	0	0	9039
**Mean** **(SD)**	**10.5** **(4.8)**	**15.5** **(5.9)**	**17.5** **(4.8)**				
**Label total**				**1357**	**1907**	**1335**	**66,468**

**Table 4:** Top-performing random undersampling (RUS)-boosted ensembles of decision trees. Target class is Total-FOG (Case 1). Mean and SD exclude nonfreezers (P04, P05, P10, P11).

	Plantar Pressure Features		IMU Features		PP-IMU Features	
	Relief-F, 5 Features, 5 Splits		mRMR, 25 Features, 5 Splits		Relief-F, 10 Features, 5 Splits	
Held out Participant	Sens (%)	Spec (%)	Sens (%)	Spec (%)	Sens (%)	Spec (%)
P01	69.7	83.7	68.2	84.0	70.0	86.0
P02	71.7	86.7	67.0	90.7	70.6	87.9
P03	68.3	89.7	54.5	96.1	61.4	92.9
P04	-	85.4	-	91.9	-	86.5
P05	-	81.3	-	88.1	-	84.6
P06	93.9	89.5	73.4	93.5	93.2	90.2
P07	72.8	80.3	34.8	92.1	68.7	78.9
P08	89.5	79.6	70.9	92.3	82.1	87.6
P09	79.7	72.5	64.6	92.2	88.6	79.7
P10	-	87.7	-	90.2	-	89.2
P11	-	79.4	-	88.3	-	79.2
**Mean** **(SD)**	**78.0** **(9.4)**	**83.2** **(5.7)**	**61.9** **(12.4)**	**91.6** **(3.4)**	**76.4** **(10.8)**	**86.2** **(4.8)**

Sens: sensitivity, Spec: specificity.

**Table 5:** Top 10 features (according to Relief-F) used in the plantar pressure and IMU (PP-IMU) features model.

Feature Rank	Feature Description
1	Dominant frequency of COP velocity in Y (AP) direction for right leg
2	Dominant frequency of COP velocity in Y (AP) direction for left leg
3	Dominant frequency of COP velocity in X (ML) direction for right leg
4	Dominant frequency of thigh acceleration in X (AP) direction for left leg
5	Number of AP COP path reversals for left leg
6	Number of AP COP path reversals for right leg
7	Minimum WT dC of COP position in Y (AP) direction for right leg
8	Dominant frequency of thigh acceleration in X (AP) direction for right leg
9	Mean energy of WT aC of COP position in Y (AP) direction for right leg
10	Mean WT aC of COP position in Y (AP) direction for right leg

AP: anterior/posterior, ML: medial/lateral, WT: wavelet transform, aC: approximation coefficient, dC: detail coefficient.

**Table 6:** Target class test cases for PP-IMU features model, using top 10 features according to Relief-F. Column headers are the label(s) included in the target class, as defined in Table 2.

	Pre-FOG (Case 2)		Pre-FOG and Pre-FOG-Transition (Case 3)		Pre-FOG-Transition (Case 4)		FOG (Case 5)	
Held out Test Participant	Sens (%)	Spec (%)	Sens (%)	Spec (%)	Sens (%)	Spec (%)	Sens (%)	Spec (%)
P01	52.5	86.0	69.5	86.0	91.6	86.0	100.0	86.0
P02	23.0	87.9	49.0	87.9	76.0	87.9	96.3	87.9
P03	37.9	92.9	57.8	92.9	79.0	92.9	88.2	92.9
P06	73.1	90.2	84.2	90.2	95.9	90.2	98.8	90.2
P07	48.8	78.9	64.5	78.9	73.2	78.9	79.9	78.9
P08	69.3	87.6	78.6	87.6	84.1	87.6	90.5	87.6
P09	81.8	79.7	87.8	79.7	96.7	79.7	100.0	79.7
**Mean (SD)**	**55.2** **(19.3)**	**86.2** **(4.8)**	**70.2** **(13.2)**	**86.2** **(4.8)**	**85.2** **(8.9)**	**86.2** **(4.8)**	**93.4** **(7.0)**	**86.2** **(4.8)**

Sens: sensitivity, Spec: specificity.

**Table 7:** Target class test cases for plantar pressure features model, using top 5 features according to Relief-F. Column headers are the label(s) included in the target class, as defined in Table 2.

	Pre-FOG (Case 2)		Pre-FOG and Pre-FOG-Transition (Case 3)		Pre-FOG- Transition (Case 4)		FOG (Case 5)	
Held out Test Participant	Sens (%)	Spec (%)	Sens (%)	Spec (%)	Sens (%)	Spec (%)	Sens (%)	Spec (%)
P01	52.5	83.7	69.2	83.7	91.0	83.7	100.0	83.7
P02	23.6	86.7	49.6	86.7	76.6	86.7	98.0	86.7
P03	43.9	89.7	64.1	89.7	85.5	89.7	100.0	89.7
P06	76.9	89.5	85.1	89.5	93.9	89.5	99.4	89.5
P07	36.7	80.3	62.9	80.3	77.4	80.3	99.2	80.3
P08	82.7	79.6	88.1	79.6	91.3	79.6	92.9	79.6
P09	70.5	72.5	78.4	72.5	90.0	72.5	100.0	72.5
**Mean (SD)**	**55.3** **(20.5)**	**83.2** **(5.7)**	**71.0** **(12.7)**	**83.2** **(5.7)**	**86.5** **(6.4)**	**83.2** **(5.7)**	**98.5** **(2.4)**	**83.2** **(5.7)**

Sens: sensitivity, Spec: specificity.

**Table 8:** Target class test cases for IMU features model, using top 25 features according to minimum-redundancy maximum-relevance (mRMR). Column headers are the label(s) included in the target class, as defined in Table 2.

	Pre-FOG (Case 2)		Pre-FOG and Pre-FOG-Transition (Case 3)		Pre-FOG- Transition (Case 4)		FOG (Case 5)	
Held out Test Participant	Sens (%)	Spec (%)	Sens (%)	Spec (%)	Sens (%)	Spec (%)	Sens (%)	Spec (%)
P01	53.5	84.0	67.9	84.0	86.7	84.0	85.7	84.0
P02	16.3	90.7	43.8	90.7	72.5	90.7	94.6	90.7
P03	31.8	96.1	49.2	96.1	67.7	96.1	94.1	96.1
P06	44.2	93.5	62.4	93.5	81.6	93.5	80.2	93.5
P07	17.8	92.1	35.5	92.1	45.4	92.1	32.9	92.1
P08	65.3	92.3	66.2	92.3	66.7	92.3	82.1	92.3
P09	50.0	92.2	62.2	92.2	80.0	92.2	100.0	92.2
**Mean (SD)**	**39.8** **(17.2)**	**91.6** **(3.4)**	**55.3** **(11.6)**	**91.6** **(3.4)**	**71.5** **(12.7)**	**91.6** **(3.4)**	**81.4** **(20.9)**	**91.6** **(3.4)**

Sens: sensitivity, Spec: specificity.

## Data Availability

The data will be made available from the authors upon reasonable request.
